# Immunotherapy and Hepatocellular Carcinoma: From Tumor-Immune Cell Interactions to Rational Therapeutic Strategies

**DOI:** 10.3390/cells15121097

**Published:** 2026-06-16

**Authors:** Kizuki Yuza, Timothy M. Pawlik

**Affiliations:** 1 Department of Surgery, The Ohio State University Wexner Medical Center and James Comprehensive Cancer Center, Columbus, OH 43210, USA; kizuki.yuza@osumc.edu; 2 Department of Gastroenterological Surgery, Yokohama City University, Yokohama 236-0004, Japan

**Keywords:** hepatocellular carcinoma, immune checkpoint inhibitor, tumor microenvironment, combination immunotherapy, resistance, cancer immunity cycle, precision immunotherapy

## Abstract

**Highlights:**

**What are the main findings?**

- HCC immunotherapy response is shaped by multiple barriers within the cancer immunity cycle, including priming failure, impaired trafficking, myeloid/stromal suppression, and metabolic-hypoxic dysfunction.
- Combination immunotherapy may be most effective when the partner therapy is biologically matched to the dominant barrier limiting checkpoint activity in a given tumor.

**What are the implications of the main findings?**

- Resistance to immunotherapy in HCC should be interpreted as tumor cell autonomous, microenvironmental, treatment-induced, and etiology-specific rather than as a single mechanism.
- A biomarker-guided and etiology-aware framework may help move HCC immunotherapy from empiric combination therapy toward precision immunotherapy.

**Abstract:**

Hepatocellular carcinoma (HCC) remains one of the leading causes of cancer-related death worldwide, and first-line systemic treatment has shifted toward immune checkpoint inhibitor (ICI)-based combinations. Response is heterogeneous, and mechanistic interpretation has lagged behind clinical practice, leaving open the question of why some tumors respond while others do not. This review uses the cancer immunity cycle as an HCC-specific scaffold to map where anti-tumor immunity fails—across priming, trafficking, suppressive myeloid or stromal, and metabolic-hypoxic barriers—and interpret combination strategies and resistance through the dominant barrier each tumor presents. ICI monotherapy rescues only specific failure points within the cycle. Combination regimens may be more effective when they are matched to one or more dominant barriers, whereas response may fail when the selected partner addresses only a secondary barrier while the dominant ecological constraint remains intact. Resistance can be similarly organized into tumor cell autonomous, microenvironmental, treatment-induced, and etiology-specific layers, with disease etiology shaping both baseline immune ecology and therapy-context vulnerability. A mechanism-based, biomarker-guided, and etiology-aware framework may help move the field from broad empiricism toward precision immunotherapy, but it should be viewed as a conceptual and translational organizing model that requires prospective testing in biomarker-stratified studies.

## 1. Introduction

### 1.1. Epidemiology and Etiology of HCC

Liver cancer remains one of the most consequential malignancies worldwide. In 2022, an estimated 865,000 new cases and 758,000 deaths were attributable to cancers of the liver and intrahepatic bile ducts, making liver cancer the sixth most commonly diagnosed cancer and the third leading cause of cancer-related death globally [1]. Hepatocellular carcinoma (HCC) accounts for more than 80% of primary liver cancers and for the majority of the mortality burden [2]. The etiologic landscape of HCC is also shifting. Although hepatitis B and C virus (HBV, HCV) infections still account for most cases worldwide, their relative contribution is declining, whereas alcohol-related liver disease and metabolic dysfunction-associated steatotic liver disease (MASLD)—including its inflammatory form, metabolic dysfunction-associated steatohepatitis (MASH)—are increasing [2]. Regardless of the initiating exposure, HCC usually develops on a background of chronic inflammation, fibrosis, and regenerative stress. This diseased hepatic milieu is not merely a substrate for carcinogenesis but also the biological context in which anti-tumor immunity must operate.

### 1.2. The Cancer Immunity Cycle as a Conceptual Framework

The cancer immunity cycle describes the sequential steps required for effective anti-tumor immunity, from tumor antigen release and presentation to T-cell priming, trafficking, tumor infiltration, and tumor cell killing [3]. In HCC, multiple steps of this cycle are disrupted by the local immune microenvironment. We therefore use the cancer immunity cycle in this review not as a generic introductory concept but as an HCC-specific scaffold to identify where anti-tumor immunity fails—across priming, trafficking, suppressive myeloid or stromal, and metabolic-hypoxic barriers—and where specific therapies may rationally intervene. Throughout, the term “dominant barrier” refers to the biological process that appears most likely—based on available molecular, spatial, translational, or clinical evidence—to limit effective anti-tumor immunity in a given context. “Barrier-matched therapy” refers to a partner therapy selected with the aim of remodeling that process. We use these terms as a conceptual and translational organizing framework rather than as prospectively validated clinical biomarkers, since the dominant barrier is rarely measured prospectively in current practice.

### 1.3. The Liver as an Immunologically Unique Organ

A defining feature of HCC is that it arises within an organ whose default immune posture is tolerogenic. Because the liver is continuously exposed to gut-derived antigens, microbial products, and metabolites through portal venous flow, it must tightly restrain excessive immune activation [4]. Kupffer cells, liver sinusoidal endothelial cells, hepatic stellate cells, and tolerogenic cytokine networks collectively maintain this anti-inflammatory state [4]. HCC therefore emerges not in immunologic isolation but within a pre-existing ecosystem that is already biased against effective anti-tumor immunity. We refer to this baseline composition and functional bias of resident immune, stromal, and vascular cells as the tumor’s immune ecology, the biological starting point from which HCC arises and within which therapy must act. Importantly, this biology is further modified by disease etiology; in particular, emerging data suggest that MASLD/MASH-related HCC may exhibit immune dysfunction that is biologically and therapeutically distinct from viral HCC [5]. Etiology, in this sense, is not only an epidemiologic variable but an immunobiologic one.

### 1.4. Evolution of Systemic Treatment and the Rise of Immunotherapy

Systemic treatment for advanced HCC was long defined by multikinase inhibitor therapy beginning with sorafenib [6]. More recently, however, first-line treatment has shifted toward immune checkpoint inhibitor (ICI)-based combinations, including both ICI plus anti-angiogenic strategies and dual-checkpoint blockade [7,8,9]. This rapid therapeutic progress has substantially changed the clinical landscape, but mechanistic interpretation has not advanced at the same pace. As a result, there remains a need for a framework that links clinical efficacy to the specific immune and stromal barriers that shape response in HCC.

### 1.5. Objective and Scope

The central thesis of this review is that tumor-immune cell interactions within the HCC tumor microenvironment (TME) determine responsiveness to immunotherapy and guide the design of rational combination strategies. We develop this thesis through a barrier-matched framework. In this framework, the tolerogenic liver environment and chronic injury provide the baseline immune ecology, whereas immune checkpoint inhibitors restore anti-tumor immunity only at selected points of failure within the cancer immunity cycle. Accordingly, combination regimens may be more effective when matched to one or more dominant barriers—including trafficking failure, priming failure, suppressive myeloid or stromal dominance, and metabolic or hypoxic effector collapse—rather than added empirically. Conversely, resistance arises when the dominant barrier is left intact, whether tumor-cell autonomous, microenvironmental, treatment-induced, or etiology-specific. Throughout, clinical trial data are interpreted not as isolated efficacy outcomes but as evidence for mechanistic remodeling of the HCC TME. This framework supports a resistance-layer-matched, biomarker-guided, and etiology-aware approach to future immunotherapy development.

Several components of this framework—including immune-excluded HCC, Wnt/β-catenin-mediated resistance, anti-VEGF-mediated vascular normalization, myeloid-stromal suppression, and etiology-dependent immune contexture—have been discussed in prior reviews. The contribution of the present review is therefore integrative rather than encyclopedic. We use the cancer immunity cycle as an HCC-specific map of immune failure; organize resistance into tumor-cell-autonomous, microenvironmental, treatment-induced, and etiology-specific layers; and interpret combination and locoregional strategies through the dominant barrier each regimen is positioned to remodel rather than through efficacy alone. We also treat etiology as an immunobiologic modifier that may shape baseline immune ecology and therapy-context vulnerability, but not as a deterministic treatment-selection criterion. This model is intended to provide a conceptual and translational structure for future biomarker-stratified studies rather than a validated clinical algorithm for current practice.

### 1.6. Literature Search Strategy

This review was designed as a narrative and mechanistically oriented synthesis rather than as a systematic review. We searched PubMed/MEDLINE from database inception through May 2026 using combinations of terms related to hepatocellular carcinoma, immunotherapy, immune checkpoint blockade, the tumor microenvironment, single-cell and spatial transcriptomic analyses, resistance, MASLD/MASH, VEGF-directed therapy, transarterial chemoembolization, stereotactic body radiation therapy, and biomarkers. Additional articles were identified through manual review of reference lists from relevant primary studies and reviews. We prioritized phase 2–3 clinical trials, translational studies with mechanistic or biomarker relevance, single-cell and spatial profiling studies, and recent reviews that provided context for rapidly evolving areas. No formal PRISMA-style protocol, quantitative synthesis, or risk-of-bias assessment was performed.

## 2. Immune Architecture of the HCC Tumor Microenvironment

**Figure 1:**
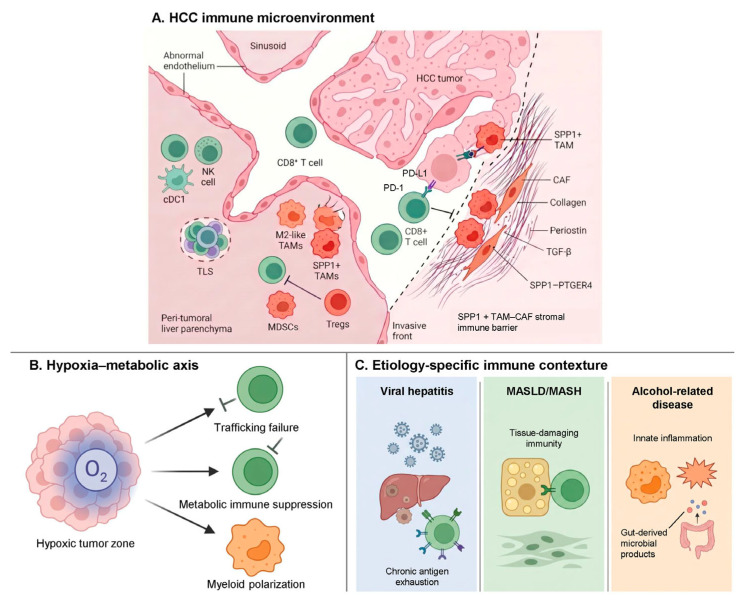
Immune architecture and etiology-dependent contexture of the hepatocellular carcinoma tumor microenvironment. Schematic overview of the pre-treatment immune ecosystem of hepatocellular carcinoma (HCC). (**A**) The sinusoidal HCC microenvironment contains anti-tumor effector populations, including CD8+ T cells, natural killer (NK) cells, conventional type 1 dendritic cells (cDC1), and tertiary lymphoid structures (TLS), together with suppressive populations such as M2-like tumor-associated macrophages (TAMs), SPP1+ TAMs, myeloid-derived suppressor cells (MDSCs), regulatory T cells (Tregs), and cancer-associated fibroblasts (CAFs). At the invasive front, SPP1+ TAMs and CAFs form a stromal–myeloid immune barrier that, together with collagen- and periostin-rich extracellular matrix and TGF-β-dominated signaling, restricts effector cell penetration into the tumor nest. The PD-1/PD-L1 axis is shown as a prototypical checkpoint interaction between an exhausted CD8+ T cell and an HCC tumor cell. (**B**) The hypoxia–metabolic axis links abnormal tumor vasculature and HIF-1α-driven signaling to three convergent consequences: impaired immune cell trafficking; metabolic immune suppression through adenosine signaling, IDO1-mediated tryptophan catabolism, and lactate accumulation; and suppressive myeloid polarization. Arrows indicate directional immune-regulatory interactions or functional consequences. (**C**) Etiology-specific immune contextures shape the baseline immune ecology of HCC: chronic antigen-driven exhaustion in HBV/HCV-related HCC; dysfunctional, tissue-damaging CD8+PD-1+CXCR6+ T-cell responses and Wnt/TGF-β-enriched cancer fields in MASLD/MASH-related HCC; and chronic innate inflammation with granulocytic MDSC enrichment in alcohol-related liver disease. Together, these components define an HCC-specific map of where the cancer immunity cycle may fail before immunotherapy is introduced. Abbreviations: CAF, cancer-associated fibroblast; cDC1, conventional type 1 dendritic cell; HCC, hepatocellular carcinoma; MDSC, myeloid-derived suppressor cell; NK, natural killer; PD-1, programmed cell death protein 1; PD-L1, programmed death-ligand 1; SPP1, secreted phosphoprotein 1; TAM, tumor-associated macrophage; TGF-β, transforming growth factor beta; TLS, tertiary lymphoid structure; Treg, regulatory T cell. This schematic is intended as a conceptual synthesis of pre-treatment HCC immunobiology rather than as a clinical decision tool.

The HCC TME is a heterogeneous ecosystem in which anti-tumor effector programs coexist with several layers of suppressive biology. Rather than enumerating individual cell types exhaustively, this section emphasizes how effector cells, suppressive cells, and non-cellular barriers together shape the dominant immune phenotype of HCC before therapy is introduced (Figure 1). Clinical and therapeutic consequences of this architecture are addressed in Section 3, Section 4 and Section 5.

### 2.1. Immune Effector Cells

Single-cell transcriptomic mapping of the human liver has resolved the cellular baseline from which HCC emerges, including hepatocyte zonation, endothelial heterogeneity, hepatic stellate cells, Kupffer cells, and the intrahepatic immune compartment [10]. Malignant transformation reorganizes this baseline architecture into a tumor ecosystem in which effector immune cells are present, but often functionally restrained.

Within HCC, CD8+ tumor-infiltrating lymphocytes display a distinct state of differentiation rather than transient inhibition, characterized by hierarchical loss of effector function and sustained expression of programmed cell death protein 1 (PD-1), T-cell immunoglobulin and mucin domain-containing protein 3 (TIM-3), and lymphocyte-activation gene 3 (LAG-3) [11]. This exhausted compartment is internally heterogeneous: a TCF1+ progenitor, or stem-like, subset retains proliferative capacity and provides the burst of responsive cells following anti-PD-1 therapy, whereas terminally exhausted CD8+ T cells contribute less to this rebound and are associated with poor response [12,13,14,15].

Natural killer (NK) cells represent a second effector arm that is particularly relevant in the liver, where they comprise nearly half of intrahepatic lymphocytes and display organ-specific phenotypic and functional features [16]. In HCC, however, NK-cell cytotoxicity is frequently constrained. Tumor-derived transforming growth factor-β1 (TGF-β1) can promote a CD96+ exhausted NK-cell phenotype marked by reduced interferon-γ (IFN-γ), tumor necrosis factor-α (TNF-α), and granzyme/perforin production. Disruption of the CD96-CD155 axis restores NK-cell cytotoxicity in preclinical HCC models, supporting the prognostic relevance and therapeutic targetability of this pathway [17].

Antigen presentation depends on a small intratumoral population of conventional type 1 dendritic cells (cDC1s), characterized in part by CD103 or CD141 expression. These cells traffic tumor antigens to draining lymph nodes through CCR7 and produce CXCL9 and CXCL10 to recruit effector T cells [18,19]. Tumors that lack this cDC1 program are therefore impaired not only in antigen presentation but also in T-cell recruitment, creating upstream failure at multiple steps of the cancer immunity cycle.

Recent neoadjuvant anti-PD-1 studies in HCC have resolved this circuitry at the tissue level, linking response to intratumoral dendritic cell–CD4+ T-helper niches that support progenitor-exhausted CD8+ T-cell differentiation, while non-response is marked by terminally exhausted CD39^hi^TOX^hi^ CD8+ T cells [20]. Effector failure in HCC is therefore not a single defect but a layered breakdown in priming, trafficking, and intratumoral differentiation, each of which becomes a separately targetable axis when combination therapy is designed.

### 2.2. Immunosuppressive Cells

Tumor-associated macrophages (TAMs) are central organizers of immune suppression in HCC. Beyond conventional M2-like polarization driven in part by colony-stimulating factor 1 (CSF1)–CSF1 receptor (CSF1R) signaling—a framework in which nomenclature has since been broadened beyond the binary M1/M2 dichotomy [21]—single-cell analyses have resolved several coexisting TAM programs, including lipid-associated TREM2+/APOE+/C1QA+ macrophages, fetal-like FOLR2+ macrophages, and invasive-front SPP1+ macrophages [22,23,24,25,26,27]. These programs are functionally relevant rather than merely descriptive: TREM2 modulation remodels the tumor myeloid landscape and enhances anti-PD-1 efficacy in preclinical models [28], whereas SPP1+ TAMs cooperate with cancer-associated fibroblasts (CAFs) to assemble an immune-excluding stromal barrier that can limit anti-PD-1 activity [29]. More recently, integrated single-cell and spatial analyses have nominated macrophage-intrinsic immune checkpoints in HCC: CD48+ TAMs are enriched in tumors, associated with immunosuppression and poor prognosis, and—through a non-canonical, CD244-independent CD48–MMP14 axis—drive protumorigenic macrophage polarization, whereas genetic deletion or antibody blockade of CD48 restores CD8+ T-cell function and synergizes with anti-PD-1 in preclinical models [30]. The functional roles of individual TAM subpopulations nonetheless remain incompletely defined and are largely supported by correlative or preclinical data.

Myeloid-derived suppressor cells (MDSCs) further reinforce this suppressive network. MDSCs include polymorphonuclear MDSCs (PMN-MDSCs) and monocytic MDSCs (M-MDSCs), which suppress T-cell function through arginase-1, inducible nitric oxide synthase (iNOS), reactive oxygen species (ROS), and immunosuppressive cytokines [31,32]. In HCC, CD14+HLA-DR-/low monocytic MDSCs can also induce CD4+CD25+Foxp3+ regulatory T cells (Tregs) through cell-contact-dependent mechanisms [33]. This interaction among suppressive populations is clinically relevant: combined depletion of Tregs, MDSCs, and PD-1+ T cells in patients with advanced HCC restores CD8+ T-cell granzyme B production to levels observed in healthy controls, indicating that these cells operate as an interdependent suppressive network rather than as parallel isolated defects [34].

Tregs contribute to immune suppression through cytotoxic T-lymphocyte-associated protein 4 (CTLA-4), interleukin-10 (IL-10), and TGF-β. The immunopathologic role of intratumoral Tregs was first established in human ovarian carcinoma, in which CD4+CD25+ Tregs were recruited to tumor sites through CCL22 produced by tumor cells and TAMs and were associated with reduced survival [35]. In HCC, intratumoral Tregs adopt an activated GITR+/ICOS+ phenotype with greater suppressive potency than circulating Tregs, and an elevated intratumoral Treg-to-CD8 ratio independently predicts poor overall survival [36,37]. The B-cell compartment is more dualistic: regulatory B cells suppress CD4+ effector responses, whereas intratumoral tertiary lymphoid structures (TLS) assemble adaptive immunity in situ and are associated with a lower risk of early post-resection recurrence [38]. Preclinical perioperative dual-ICI data extend this dualism, as B-cell depletion abrogated anti-PD-1/CTLA-4 efficacy in mouse HCC and implicated a B-cell-dependent component of effective anti-tumor immunity [39]. Together, these data indicate that immune regulation in HCC is not governed by independent cell types but by spatially organized and mutually reinforcing networks that shape effector-cell entry, activation, and persistence.

### 2.3. Hypoxia-Driven Immune Dysfunction

Rapid tumor growth and abnormal vascular remodeling generate regional hypoxia that stabilizes hypoxia-inducible factor-1α (HIF-1α) and activates transcriptional programs that upregulate vascular endothelial growth factor (VEGF), CXCL12, and adenosine-generating ectonucleotidases [40]. The resulting irregular vasculature impairs both immune-cell trafficking and drug delivery, creating a physical and functional barrier to effective anti-tumor immunity. Hypoxia also acts directly on effector lymphocytes: combined with persistent antigenic stimulation, hypoxic stress disrupts peroxisome proliferator-activated receptor-γ coactivator-1α (PGC-1α)-dependent mitochondrial reprogramming, increases reactive oxygen species, and accelerates CD8+ T-cell exhaustion [41]. Hypoxia therefore links three major failures of the HCC immune microenvironment—impaired trafficking, metabolic stress, and terminal T-cell exhaustion—and provides the biological rationale for vascular normalization strategies discussed in Section 4.

### 2.4. Metabolic Immune Suppression

Metabolic competition further constrains anti-tumor immunity in HCC. Tumor cells and infiltrating lymphocytes compete for glucose within the tumor niche, and tumor-imposed glucose restriction dampens T-cell mechanistic target of rapamycin (mTOR) activity, glycolytic capacity, and IFN-γ production, thereby limiting effector function even when antigen recognition is preserved [42]. Lactate accumulation adds a second metabolic barrier by blunting T- and NK-cell effector activity through nuclear factor of activated T cells (NFAT) downregulation and reduced IFN-γ production [43]. Amino acid depletion adds a third layer of suppression: tryptophan catabolism through indoleamine 2,3-dioxygenase 1 (IDO1) and arginine depletion through arginase-1 reduce local T-cell function, with arginase-1 representing a hallmark suppressive mechanism of CD14+HLA-DR-/low monocytic MDSCs in HCC [33]. In parallel, extracellular adenosine triphosphate (ATP) is converted by CD39 and CD73 into adenosine, which signals through A2A receptors on effector cells and constitutes a distinct, pharmacologically tractable suppressive node now being explored in combination with checkpoint blockade [44,45]. Thus, metabolic suppression is not separate from hypoxia or myeloid biology, rather representing the biochemical layer through which the suppressive HCC ecosystem disables otherwise targetable immune cells.

### 2.5. Structural and Stromal Barriers

HCC almost invariably arises on a fibrotic background, and single-cell mapping of human cirrhosis has demonstrated that scar-associated macrophage, endothelial, and collagen-producing mesenchymal populations form pro-fibrogenic niches before malignant transformation [46]. These cirrhotic programs overlap conceptually with the onco-fetal endothelial and macrophage niches identified in HCC, suggesting a continuum from chronic liver injury to malignant TME reprogramming [26]. Spatial and single-cell profiling has further resolved this program, identifying co-localized POSTN+ extracellular-matrix CAFs, FOLR2+ macrophages, and PLVAP+ endothelial cells as an onco-fetal niche associated with early relapse and differential response to immunotherapy [47].

Resident macrophage identity in the liver is also shaped by the local tissue niche. Single-cell mapping of normal human liver has resolved distinct intrahepatic macrophage populations with inflammatory and immunoregulatory profiles [48], while depletion-repopulation studies have noted that recruited monocytes can acquire Kupffer cell identity through cooperative signals from hepatic stellate cells, sinusoidal endothelial cells, and hepatocytes [49,50]. More broadly, tissue-resident macrophage subsets occupy specialized perivascular and perineural niches that contribute differentially to fibrotic remodeling [51]. These observations imply that fibrotic and tumor-driven remodeling of the hepatic niche directly reshapes the identity and function of macrophages within established HCC.

Within established HCC, CAFs are not a uniform structural compartment. Cross-cancer single-cell studies have resolved myofibroblastic, inflammatory, and antigen-presenting CAF states [52,53], while HCC-specific analyses have identified a POSTN+ CAF subpopulation that physically excludes CD8+ T cells and recruits SPP1+ TAMs through IL-6/STAT3 signaling [54]. Together with spatial transcriptomic evidence that SPP1+ macrophage–CAF assemblies form a tumor immune barrier limiting anti-PD-1 efficacy, these data indicate that stromal remodeling is both mechanical and immunologic [29,54]. The tumor vasculature can itself be actively immunosuppressive: a CXCL12+ tumor-associated endothelial cell (TEC) subpopulation impedes differentiation of naïve CD8+ T cells into cytotoxic effectors and recruits MDSCs, and a bispecific antibody co-targeting CXCL12 and PD-1 enhanced anti-tumor immunity in preclinical HCC [55]. The structural barrier is therefore not a passive scaffold but an active determinant of immune-cell access and effector function in HCC, providing the mechanistic rationale for the stromal-, vascular-, and myeloid-remodeling combinations examined in Section 4.

### 2.6. Immune Checkpoint and Immune-Exclusion Programs

The suppressive ecosystem described above converges on checkpoint-ligand and immune-exclusion programs. HCC tumors can express programmed death-ligand 1 (PD-L1), Galectin-9, CD155, and TGF-β-dominant transcriptional programs, but these signals should be interpreted as downstream outputs of cellular, stromal, hypoxic, and metabolic suppression rather than as isolated biomarkers [56,57]. This distinction is mechanistically important. Checkpoint blockade can release inhibitory signaling in tumors with pre-existing immune engagement, but it cannot by itself restore absent priming, reverse stromal exclusion, or correct hypoxia-driven metabolic dysfunction. Checkpoint and immune-exclusion programs therefore represent the most pharmacologically tractable face of a deeper suppressive architecture in HCC, explaining both the clinical promise of ICI monotherapy and its limits when other barriers dominate, a distinction developed in Section 3.

### 2.7. Immune Phenotype Classification

These immune programs can be organized into immune-inflamed, immune-excluded, and immune-desert phenotypes that help explain response heterogeneity under ICI-based therapy [58]. In HCC, transcriptomic analyses have identified an immune-specific class marked by cytolytic activity, PD-1/PD-L1 expression, and interferon signaling, as well as an exhausted TGF-β-enriched subtype, indicating that even “inflamed” tumors may contain both effector and suppressive programs [56]. More recent molecular classifications further divide HCC into immune-active, immune-exhausted, immune-like, immune-intermediate, and immune-excluded states, linking immune phenotype to CTNNB1 mutation status, TP53-driven chromosomal instability, and broader tumor biology [59]. Clinically, atezolizumab-plus-bevacizumab-treated cohorts have also resolved reproducible phenotypic groups associated with progression pattern, liver function, and survival, supporting the idea that immune contexture is not only molecularly measurable but clinically relevant [60]. Complementary single-cell and spatial studies have begun to translate these phenotypes into outcome-associated classifiers. In atezolizumab-plus-bevacizumab-treated cohorts, single-cell-derived signatures identified distinct benefit patterns, including an immune-mediated response marked by CD8+ effector T cells and CXCL10+ macrophages, an angiogenesis-associated response marked by low neuropilin-1, and a resistant subset enriched for immunosuppressive myeloid cells and Notch activation [61]. In parallel, spatial single-cell profiling defined immune-depleted, compartmentalized, and immune-enriched immunotypes based on CD8+ T-cell abundance and parenchymal–stromal distribution, with the immune-enriched pattern associated with longer progression-free survival under ICI therapy [62]. Thus, immune phenotype classification provides an integrative readout of the cellular and non-cellular barriers described in this section and helps frame the therapeutic logic of checkpoint blockade and combination therapy.

### 2.8. Etiology-Dependent Immune Contexture

A distinctive feature of HCC among solid tumors is that etiology systematically reshapes the immune ecosystem. In HBV-related disease, chronic viral antigen persistence promotes a mitochondrial-dysfunction-centered exhaustion program in HBV-specific CD8+ T cells, creating an immune infiltrate that is inflamed yet functionally constrained [63]. HCV-related HCC similarly shows chronic PD-1 and TIM-3 upregulation on T cells, together with impaired major histocompatibility complex (MHC) and co-stimulatory signaling on antigen-presenting cells [64].

MASLD/MASH-related HCC is biologically distinct. Molecularly, MASH-related HCC carries higher rates of ACVR2A mutations, a steatohepatitis-associated mutational signature, and enrichment for the Wnt/TGF-β proliferation subclass, consistent with an immunosuppressive pro-carcinogenic field rather than a classical inflamed phenotype [65]. Functionally, aberrantly activated resident-like CD8+PD-1+CXCR6+ T cells accumulate within the steatotic liver and drive chronic tissue damage more readily than effective tumor clearance [5,66]. This biology has been proposed as one possible contributor to heterogeneous ICI benefit in non-viral HCC; however, the proposal remains controversial, and differences in clinical response across etiologies appear modest in many datasets.

Alcohol-related liver disease adds a third pattern, in which acetaldehyde-driven DNA adducts, reactive oxygen species, lipid peroxidation, and impaired innate immunity shape the pre-malignant immune ecology, often in synergy with diabetes, obesity, or viral hepatitis [67]. Across non-viral etiologies, microbe-associated molecular patterns and bacterial metabolites derived from a disrupted gut barrier further tune hepatic inflammatory tone through the gut-liver axis [68,69]. Etiology in HCC should therefore be treated not as a background descriptor but as the baseline immune ecology that defines each tumor’s starting point for therapy; how this ecology becomes manifest as therapy-context resistance is taken up in Section 5.4. At the same time, etiology should not currently be used as a standalone biomarker to select or withhold ICI-based therapy: clinical evidence for differential ICI benefit across etiologies remains heterogeneous, and high-resolution spatial profiling indicates that HCC immune architecture is not strictly determined by underlying etiology [62].

## 3. Immune Checkpoint Inhibitors: Mechanistic Basis and Clinical Translation

### 3.1. PD-1/PD-L1 Pathway

PD-1 is expressed on T cells after priming, and ligation by PD-L1 or PD-L2 recruits the phosphatase SHP-2, which dephosphorylates proximal T-cell receptor-signaling components and attenuates effector output [3,57]. Blockade with anti-PD-1 or anti-PD-L1 antibodies therefore works preferentially in tumors that already contain pre-existing T-cell immunity, reactivating TCF1+ stem-like precursor CD8+ T cells that can self-renew and repopulate the effector pool rather than rescuing terminally exhausted cells [58]. In HCC, this biology is consistent with the clinical pattern observed across PD-1-directed studies. Nivolumab in CheckMate 459 and pembrolizumab in KEYNOTE-224/240 produced durable responses in subsets of patients, although phase 3 confirmation was limited in Western cohorts [70,71,72]. The Asian phase 3 KEYNOTE-394 trial subsequently confirmed a survival benefit for pembrolizumab versus placebo in the second-line setting, supporting a reproducible but context-dependent role for PD-1 monotherapy in HCC [73]. Taken together, PD-1/PD-L1 monotherapy can rescue HCC tumors that are immunologically primed but restrained by this axis, while leaving upstream deficits in priming, trafficking, or immune exclusion uncorrected.

### 3.2. CTLA-4 Pathway

CTLA-4 restrains T-cell activation during priming by competing with CD28 for CD80/CD86 on antigen-presenting cells. CTLA-4 is also constitutively expressed at high levels on Tregs, where it contributes to contact-dependent suppression [3,57]. CTLA-4 blockade therefore has two mechanistically distinct effects in HCC: it broadens the priming of tumor-reactive T cells in lymphoid tissues, and it may reduce the Treg-mediated brake on effector responses within the TME. Tremelimumab was the first CTLA-4-directed agent with documented activity in HCC and provided the mechanistic backbone for subsequent dual ICI development [57].

### 3.3. Why Dual Checkpoint Blockade Can Work in HCC

Because CTLA-4 and PD-1 act at different phases of the anti-tumor immune response, their combined blockade is mechanistically expected to expand clonal diversity, promote epitope spreading, and rescue effector function rather than merely adding two inhibitory antibodies [3,57,58]. This logic is embedded in the HIMALAYA STRIDE regimen, in which a single priming dose of tremelimumab is followed by continued durvalumab, with the aim of augmenting priming without the toxicity burden of sustained CTLA-4 exposure [8]. HIMALAYA provided clinical proof-of-principle that dual checkpoint blockade can improve survival in unresectable HCC [8]. The concept was further extended by CheckMate 9DW, in which nivolumab plus ipilimumab improved overall survival versus lenvatinib or sorafenib, supporting the broader principle that layered checkpoint blockade is not confined to a single agent pair [9].

### 3.4. Clinical Implementation Across Disease Stages

Across advanced first-line disease, several mechanistic classes of ICI-based regimens now coexist, each best understood according to the barrier of the cancer immunity cycle it primarily remodels. IMbrave150 established ICI plus anti-VEGF therapy as a reference strategy in which checkpoint blockade is paired with vascular normalization and partial myeloid reprogramming [7,74]. HIMALAYA and CheckMate 9DW represent dual-ICI approaches that engage priming and effector phases simultaneously, whereas CARES-310 and ORIENT-32 demonstrate that the ICI-plus-anti-angiogenic concept generalizes across compounds and geographic or etiologic contexts [8,9,75,76]. Trial-level efficacy estimates are consolidated in Table 1; in the body, the relevant question is which immune-cycle bottleneck each combination is biologically positioned to remodel.

The perioperative setting raises a distinct question: whether checkpoint blockade can engage the intact tumor as an in situ antigen source before resection, when tumor antigen availability, nascent priming, micrometastatic disease control, and recurrence biology converge within a single therapeutic window. Randomized perioperative nivolumab with or without ipilimumab demonstrated meaningful major pathologic response and acceptable feasibility in resectable HCC, supporting the principle that checkpoint engagement can be moved earlier in the disease course [77]. Long-term follow-up of perioperative dual ICI in a Taiwan phase 2 cohort extended this signal and implicated intratumoral TLS signatures and B-cell-dependent immunity as candidate determinants of response [39]. These findings are mechanistically consistent with HCC-specific evidence that intratumoral dendritic cell–CD4+ T-helper niches license CD8+ T-cell differentiation following PD-1 blockade, suggesting that the perioperative interval may provide a tissue context conducive to productive priming [20].

Adjuvant strategies, by contrast, have proved more conditional. IMbrave050 reported an early recurrence-free survival benefit for adjuvant atezolizumab plus bevacizumab after curative resection or ablation in high-risk patients [78]. With longer follow-up, however, this benefit was not sustained and overall survival remained immature, such that the totality of evidence does not currently support adjuvant atezolizumab plus bevacizumab as a routine standard of care [59,79]. Instructive negative trials in advanced disease reinforce the same principle. COSMIC-312 and LEAP-002 each had biologically plausible rationales, yet neither produced a clear overall-survival benefit versus its comparator [80,81]. These outcomes may reflect incomplete or mismatched TME remodeling, but this interpretation is not the only explanation and may be secondary to trial-level factors, including active-comparator efficacy, statistical design and alpha allocation, patient selection, baseline liver function, etiologic composition, toxicity and treatment discontinuation, and subsequent therapies. Mechanistic interpretation should therefore be regarded as a contributory lens rather than the sole or definitive explanation for these results. Nevertheless, the mechanistic lesson is that the simple presence of an ICI is insufficient; what matters is whether the regimen remodels the dominant barriers—trafficking failure, defective priming, and suppressive myeloid or stromal programs—in a given tumor. Table 1 summarizes these trials so that this barrier-matched logic can be read across regimens rather than obscured by sequential trial narration.

## 4. Cellular Mechanisms of Combination Therapies

### 4.1. ICI + Anti-VEGF/TKI: Vascular Normalization and Myeloid Remodeling

The dominant barriers addressed by anti-angiogenic combinations are trafficking failure and suppressive myeloid dominance. HCC tumors typically develop chaotic, hyperpermeable vasculature that limits effector lymphocyte access and drug delivery, while VEGF-centered signaling promotes TAM polarization and MDSC accumulation toward an immunosuppressive state [82,83,84]. VEGF blockade can transiently restore pericyte coverage, improve intratumoral perfusion, and reduce endothelial dysfunction, opening a vascular-normalization window in which immune-cell access becomes more permissive and intratumoral CD8+ T-cell infiltration may increase while Treg recruitment is attenuated [82,84].

Although anti-VEGF antibodies and tyrosine kinase inhibitors (TKIs) converge on this anti-angiogenic axis, the immunologic effects are not identical. Antibody-based VEGF blockade most directly exemplifies vascular normalization, whereas multikinase inhibitors such as lenvatinib, cabozantinib, and rivoceranib additionally modulate VEGFR2-, c-Met-, and related kinase-driven signaling within myeloid and stromal compartments, favoring a shift toward more inflammatory macrophage states, reduced suppressive myeloid tone, and improved dendritic-cell maturation [84]. The shared biology of IMbrave150, CARES-310, and ORIENT-32 is therefore the concurrent correction of a trafficking-dominant barrier and a myeloid-dominant barrier, rather than the inclusion of an ICI per se [7,74,75,76]. Vascular normalization and immune remodeling are not accessory effects of these regimens; they are central to why checkpoint blockade becomes more effective in HCC.

### 4.2. ICI + Chemotherapy/Hepatic Arterial Infusion: Immunogenic Cell Death and Priming Rescue

The dominant barrier addressed by chemotherapy-based and hepatic arterial infusion chemotherapy (HAIC) combinations is priming failure. Platinum-based regimens, delivered systemically or regionally, can promote immunogenic cell death, in which dying tumor cells expose calreticulin and release ATP and high-mobility group box 1 (HMGB1) [83,84]. These damage-associated molecular patterns (DAMPs) license dendritic cells, enhance antigen uptake and cross-presentation, and strengthen priming of tumor-specific CD8+ T cells, the effector pool that PD-1/PD-L1 blockade is intended to reinvigorate [84]. In HCC, this mechanism is especially attractive in tumors with high intrahepatic burden or poorly inflamed lesions, in which priming failure may be as important as terminal T-cell dysfunction. Oxaliplatin is of particular interest because it has been linked to immunogenic cell death biology [84]. Chemotherapy-mediated cytoreduction may lessen local suppressive signaling by decreasing tumor bulk, reducing necrotic-hypoxic burden, and altering suppressive myeloid and stromal elements within the tumor bed.

Early studies of HAIC plus anti-PD-(L)1 therapy have reported encouraging activity in selected patients with liver-dominant disease not well suited to transarterial chemoembolization (TACE); however, mature phase 3 evidence remains limited, and the precise clinical niche of HAIC-based immunotherapy combinations is still evolving [84]. Mechanistically, these regimens are best understood as strategies to restore antigen release and cross-priming in tumors where checkpoint blockade alone may be attempting to rescue T cells that were never adequately primed in the first place.

### 4.3. ICI + Transarterial Chemoembolization: In Situ Vaccination in a Residual Hypoxic Bed

TACE addresses priming failure through antigen release, but generates a parallel suppressive challenge by intensifying local hypoxia, perturbing the residual vascular bed, and increasing PD-L1 expression on surviving malignant and stromal cells; the post-TACE lesion may therefore become simultaneously more antigenic and more suppressive [83,85]. This duality is central to understanding why TACE is a rational but incomplete immune primer when used alone.

The most instructive randomized data support this interpretation. In EMERALD-1, durvalumab plus bevacizumab added to TACE prolonged progression-free survival, whereas durvalumab added to TACE without bevacizumab did not [86]. The contrast suggests that antigen release after TACE must be coupled with vascular and myeloid remodeling of the residual tumor bed if checkpoint blockade is to achieve durable benefit. In LEAP-012, lenvatinib plus pembrolizumab plus TACE prolonged progression-free survival versus TACE alone, although overall survival benefit was not established at the primary report [87]. Both regimens were associated with increased grade 3–4 toxicity relative to TACE alone, reinforcing the need for balanced interpretation [85].

The common theme across these studies is that TACE provides local antigen release and inflammatory priming, but its immune consequences are shaped by the residual ecological state it leaves behind. From a treatment-design perspective, locoregional therapy in HCC is therefore best understood not merely as local cytoreduction but as a controlled mode of antigen exposure whose immunologic effect depends on whether the systemic partner remodels the vascular, myeloid, and hypoxic context that persists after embolization.

### 4.4. ICI + SBRT/Ablation: Radiation-Primed Immune Activation and Antigen Diversification

Stereotactic body radiation therapy (SBRT) addresses priming failure through a mechanistically distinct route: radiation-induced immunogenic tumor cell death generates cytosolic double-stranded DNA that activates the cGAS-STING pathway, stimulates type I interferon signaling, and enhances dendritic cell activation and tumor antigen presentation [83,84]. Radiation may also upregulate MHC class I expression and broaden the antigenic repertoire available for immune recognition, increasing the likelihood that poorly inflamed lesions acquire a more immunogenic phenotype [83]. SBRT is thus not simply a local cytoreductive tool but a biologic primer that may link focal tumor injury to broader immune activation.

Early clinical data support this framework, although mature evidence remains limited. The PEMRAD phase 2 trial reported a meaningful objective response rate for pembrolizumab combined with SBRT in sorafenib-pretreated advanced HCC, including responses among patients with macrovascular invasion, a population in whom effective systemic options have historically been limited [88]. RTOG 1112, which paired SBRT with sorafenib rather than an ICI, provides complementary support for the broader principle that focal radiation can be integrated with systemic therapy as a disease-control and immune-modifying strategy in locally advanced HCC [89]. Its relevance here is therefore mechanistic rather than regimen-specific: it supports SBRT as a systemic-therapy partner in advanced HCC, while direct evidence for SBRT plus ICI remains less mature.

SBRT and TACE occupy distinct biologic and anatomic niches. TACE relies on arterial occlusion and ischemic necrosis, whereas SBRT may be especially useful in lesions with macrovascular invasion, portal vein tumor thrombus, or anatomy less amenable to embolization. Thermal and chemical ablation create similar opportunities for local antigen exposure, although with different temporal and spatial cell death kinetics [83]. From a treatment design perspective, focal therapy in HCC is best understood not as a set of interchangeable cytoreductive options but as a configurable mode of immune priming; the choice among embolization, radiation, and ablation should follow the priming deficit, disease anatomy, and residual microenvironmental barrier each modality most directly addresses.

### 4.5. A Unifying Principle for Combination Design

These regimens succeed to the extent that they correct one or more dominant failures in the HCC cancer immunity cycle. Trafficking failure can be addressed by anti-VEGF-mediated vascular normalization; priming failure by chemotherapy-, HAIC-, TACE-, or SBRT-driven antigen release; suppressive myeloid or stromal dominance by VEGF blockade and TKI-mediated reprogramming; and metabolic or hypoxic effector collapse by improved perfusion in previously hypoxic tumors [3,57,83,84]. The common logic is therefore not that more therapy is better, but that each partner must be biologically matched to the dominant barrier operating in that tumor.

This framework also clarifies why simply adding an ICI to another modality is not inherently synergistic. Clinical benefit is most likely when the second agent addresses a bottleneck that checkpoint blockade alone cannot overcome: poor trafficking, inadequate priming, myeloid suppression, stromal exclusion, or hypoxia-driven dysfunction. Response may fail even when checkpoint blockade is pharmacologically active if the selected partner corrects only a secondary barrier while the dominant ecological constraint remains intact. Many failures of combination therapy in HCC should therefore be considered not as failure of checkpoint inhibition per se but as incomplete or mismatched remodeling of the TME, the same logic that organizes the resistance categories examined in Section 5.

## 5. Resistance Mechanisms

### 5.1. Intrinsic Resistance (Tumor-Cell Autonomous)

Intrinsic resistance in HCC is best understood as failure of the tumor cell to generate or sustain the conditions required for effective immune priming and effector engagement. The prototypical example is Wnt/β-catenin pathway activation, typically driven by CTNNB1 or AXIN1 alterations, which defines a major molecular subset of HCC and is associated with immune exclusion rather than absence of inflammation alone [65,90]. In this setting, the central defect is not simply low inflammation but defective recruitment of the dendritic cell populations required for productive T-cell priming.

Mechanistic work has established this point directly. In a hydrodynamic-injection murine HCC model, constitutive β-catenin activation downregulated chemokine programs including CCL5, CCL20, and CXCL1; impaired recruitment of CD103+ dendritic cells; and thereby prevented priming of tumor-specific CD8+ T cells; re-expression of CCL5 restored immune surveillance, indicating that chemokine-driven failure of immune-cell trafficking is a core operative mechanism [91]. Thus, Wnt/β-catenin-mediated resistance in HCC is not merely a genomic correlate of non-response but a direct tumor-cell program that creates an immune-desert state upstream of checkpoint blockade.

Clinical observations are concordant with this biology. In a prospective MSK-IMPACT cohort of patients with advanced HCC treated with anti-PD-(L)1 therapy, none of the patients with activating WNT/β-catenin alterations derived clinical benefit, whereas 53% of individuals without such alterations did; importantly, WNT status did not predict outcomes with sorafenib, supporting its interpretation as a predictive rather than simply prognostic biomarker for ICI responsiveness [90]. Additional tumor-cell-autonomous resistance programs include loss of antigen presentation machinery through large copy-number events and the generally low tumor mutational burden characteristic of HCC, both of which constrain neoantigen visibility and limit the substrate on which checkpoint reactivation can act [59,92]. Together, these findings indicate that some HCCs fail to respond because the malignant cell itself establishes an ecosystem in which immune recognition is defective before therapy even begins.

### 5.2. Extrinsic Resistance (Microenvironment-Mediated)

Even when tumor cells remain antigenic and potentially targetable, response may still fail because the surrounding microenvironment preserves suppressive dominance despite treatment. In HCC, this ecological resistance is maintained by interlocking myeloid, stromal, vascular, and metabolic barriers that can blunt the functional consequences of checkpoint reinvigoration. In other words, T cells may be reactivated pharmacologically yet remain spatially excluded, metabolically impaired, or rapidly suppressed once they enter the tumor bed.

Several microenvironmental pathways appear particularly important. TAMs, MDSCs, and Tregs collectively generate a soluble suppressive milieu enriched in IL-10, TGF-β, arginase-1, reactive oxygen species, and adenosine-generating pathways, thereby neutralizing cytotoxic lymphocytes even after PD-1/PD-L1 inhibition has been introduced [93,94]. In parallel, CAF-rich stromal niches and invasive-front myeloid-stromal assemblies create structured regions of immune exclusion. The SPP1+ macrophage–CAF tumor immune barrier introduced in Section 2.5 is mechanistically central. Hypoxia within the tumor core drives SPP1 expression, and the resulting extracellular matrix remodeling actively restricts cytotoxic T-cell penetration even when checkpoint signaling is pharmacologically blocked [29]. Importantly, this barrier is therapeutically actionable, as SPP1 blockade or macrophage-specific Spp1 deletion in murine HCC reduced CAF infiltration, increased intratumoral cytotoxic T cells, and enhanced anti-PD-1 efficacy [29]. These barriers help explain why some tumors remain non-responsive despite evidence of immune engagement in other compartments.

Hypoxia and vascular dysfunction reinforce this resistant state. HIF-1α stabilization promotes VEGF, CXCL12, and adenosine-generating pathways, thereby impairing immune-cell trafficking while simultaneously favoring suppressive myeloid accumulation and polarization [82]; consistent with this, a CXCL12+ tumor-associated endothelial cell program has been implicated in immune resistance in HCC [55]. Fibrotic extracellular matrix, collagen-rich CAF programs, and periostin-containing stromal networks further restrict T-cell penetration into tumor nests, sustaining the immune-excluded phenotype that characterizes a substantial fraction of primary ICI non-responders [83,93]. The key point is that these processes do not operate as parallel minor obstacles. Rather, they form an interdependent architecture of ecological resistance that can persist even when checkpoint blockade is biologically active.

### 5.3. Adaptive Resistance (Treatment-Induced)

Resistance in HCC is not only pre-existing; it can also emerge under therapeutic pressure after an initial immune response has been partially restored. Adaptive resistance refers to the dynamic reprogramming that follows immune engagement, in which tumor and microenvironmental compartments respond to ongoing cytotoxic pressure by reinstating inhibitory signaling through alternative pathways. In this context, treatment does not fail because nothing changed but because the system changed in a direction that re-established immune restraint.

A central feature of this process is compensatory checkpoint upregulation. Persistent T-cell activation during PD-1 pathway blockade can induce increased expression of LAG-3, TIM-3, TIGIT, and related inhibitory receptors on effector and exhausted T-cell populations, thereby restoring a functional brake that the original therapeutic antibody does not address [84]. At the same time, immune-active tumors may undergo cytokine and stromal rewiring, including reinforcement of TGF-β-associated excluded states and persistence of transcriptional programs linked to immune exhaustion [56]. Although much of the highest-resolution evidence for adaptive remodeling comes from other tumor types, including melanoma and murine models of checkpoint response, these studies show that response and resistance to checkpoint blockade are accompanied by coordinated shifts in memory-like CD8+ T-cell states, exhausted T-cell programs, and intratumoral myeloid polarization [95,96]. Similar dynamic plasticity is likely relevant in HCC, where progenitor-exhausted CD8+ T cells and stromal-myeloid reprogramming have been described in the neoadjuvant cohorts discussed in Section 2.1 and Section 2.2. Thus, adaptive resistance should be understood not as failure of the initial target but as selective, pressure-driven remodeling of the inhibitory landscape.

Empirically, this caution is reinforced by clinical translational data. For example, a phase 3 study evaluating anti-TIGIT added to atezolizumab plus bevacizumab in advanced HCC did not meet its primary endpoint, underscoring that compensatory checkpoints are not automatically dominant or readily druggable in all tumors [97]. Adaptive resistance therefore functions both as a biological mechanism and a conceptual warning. Resistance-directed combinations will succeed only when the emergent inhibitory pathway is genuinely a dominant barrier rather than an epiphenomenon of ongoing immune activation, a constraint that frames the next-generation checkpoint strategies revisited in Section 6.2.

### 5.4. Etiology-Specific Resistance

The immune failure modes of HCC are further shaped by disease etiology. Section 2.8 introduced viral-, MASLD/MASH-, and alcohol-related HCC as distinct baseline immune ecologies that define each tumor’s starting point for therapy; this subsection addresses how those ecologies may become manifest as therapy-context resistance because the barriers left untouched by checkpoint blockade may differ across etiologic backgrounds. Etiology is therefore not merely a background descriptor but a variable that conditions how resistance is constructed under treatment pressure.

The clearest example is MASLD/MASH-related HCC. In this setting, aberrantly activated resident-like CD8+PD-1+CXCR6+ T cells appear capable of driving chronic tissue injury more readily than effective tumor clearance, raising the possibility that PD-1 blockade may, in some patients, intensify a dysfunctional auto-aggressive program rather than restore productive anti-tumor immunity [5,66]. Non-viral HCC is also enriched for molecular subclasses marked by Wnt/TGF-β-associated signaling and relatively cold immune contexture, which may reinforce resistance through both tumor-intrinsic and microenvironmental routes [65]. These mechanisms remain hypothesis-generating, and the magnitude of any etiology-specific difference in clinical ICI benefit remains uncertain. These observations help explain why an apparently inflamed liver does not necessarily translate into a tumor that is immunologically responsive.

By contrast, HBV-related HCC often displays chronic antigen-driven exhaustion programs that may remain at least partially rescuable by checkpoint inhibition, while other viral contexts may preserve distinct immune vulnerabilities not shared by metabolically driven disease [93]. The practical implication is that the “barrier-left-untouched” model proposed in Section 4.5 operates not only across mechanistic levels but also across etiologic backgrounds. Some tumors fail because priming is absent, others because exclusion dominates, others because suppressive rewiring emerges during therapy, and still others because the underlying liver disease biases the immune system toward tissue-damaging rather than tumor-clearing responses. This etiology-conditioned view of resistance provides the rationale for the resistance-layer-matched and biomarker-guided strategies developed in Section 6.

## 6. Future Perspectives

### 6.1. Overview

Immunotherapy has transformed the treatment landscape of HCC, yet clinical benefit is determined less by the nominal regimen selected than by the immune architecture of the individual tumor. In this review, we used the cancer immunity cycle not as a generic conceptual frame but as an HCC-specific map of where anti-tumor immunity fails: at the level of priming, trafficking, effector rescue, stromal exclusion, metabolic suppression, and etiology-conditioned immune dysfunction. From this perspective, the major contribution of modern combination therapy is not simply additive efficacy but active remodeling of the dominant biological barrier that limits checkpoint activity in a given tumor.

This same framework also explains why rational combinations do not succeed universally. Response fails when the selected regimen leaves the dominant barrier intact, whether that barrier is tumor-cell autonomous, microenvironmental, treatment-induced, or etiology-specific. Future progress will therefore likely require closer alignment between therapies that target specific resistance mechanisms and biomarkers that identify which resistance layer is most relevant in each patient. Table 2 summarizes this framework by linking dominant barriers to representative biology or biomarkers, therapeutic rationale, example strategies, and current clinical applicability.

### 6.2. Emerging Therapeutic Strategies

Emerging therapeutic classes can be understood as attempts to intervene on specific resistance layers defined in Section 5 rather than as generic extensions of the current ICI era.

Adaptive resistance is the focus of next-generation checkpoint strategies. Antibodies directed against LAG-3, TIM-3, TIGIT, and related inhibitory receptors are conceptually attractive because these pathways may emerge after PD-1/PD-L1 blockade and restore immune restraint. Early phase 3 results with an anti-TIGIT-containing triplet have been disappointing, however, underscoring that not every induced checkpoint becomes a dominant and druggable barrier in clinical practice [97]. The lesson is not that adaptive checkpoints are irrelevant but that their therapeutic value depends on better biological matching to the tumors in which they truly govern immune escape.

Extrinsic suppressive circuits define another therapeutic direction. TGF-β- and adenosine-centered programs reinforce immune exclusion, stromal restraint, and metabolic dysfunction, often limiting T-cell activity even when lymphocytes are present. Agents targeting the glycoprotein A repetitions predominant (GARP)-TGF-β1 axis, including livmoniplimab in combination with budigalimab, exemplify efforts to weaken a suppressive stromal-immune network while preserving checkpoint rescue [98]. Blockade of the CD39–CD73–adenosine axis similarly aims to relieve a metabolically hostile microenvironment that constrains effector function [84,93]. Macrophage-intrinsic checkpoints add a further myeloid-directed option, as CD48 on tumor-associated macrophages promotes suppression through a non-canonical, CD244-independent mechanism, whereas CD48 blockade enhances anti-PD-1 activity in preclinical HCC [30]. These approaches target resistance at the ecological level rather than solely at the level of lymphocyte inhibition, and they remain largely investigational, in contrast to the established first-line immunotherapy combinations discussed in Section 3.

Priming failure is addressed by strategies that bypass or augment endogenous antigen presentation. Bispecific T-cell engagers and cell-based therapies, including glypican-3 (GPC3)-directed chimeric antigen receptor T-cell (CAR-T) constructs, redirect cytotoxic immunity toward tumor cells with less dependence on the quality of endogenous priming [97,98]. Oncolytic viruses such as VG161 and therapeutic vaccine platforms similarly aim to increase tumor immunogenicity upstream of checkpoint engagement by broadening antigen exposure and inflammatory activation [99]. The key future direction will likely be combination design rather than monotherapy development. The most effective next-generation regimens will pair established ICI backbones with agents chosen to correct the dominant untreated barrier in each tumor.

### 6.3. Toward Biomarker-Driven Precision Immunotherapy

The parallel challenge is to identify biomarkers that can help match patients to barrier-specific combinations from which they may benefit. In HCC, currently available biomarkers such as PD-L1 expression and tumor mutational burden remain insufficient because they incompletely capture the ecological structure of the TME [59]. More promising tumor- and tissue-based frameworks include immune phenotype classification, molecular programs associated with immune exclusion such as CTNNB1 activation, and spatially organized suppressive barriers such as the SPP1+ TAM-CAF niche [90,92]. Recent single-cell and spatial studies extend this concept by identifying candidate classifiers of ICI benefit and resistance, including myeloid/Notch-associated resistance programs, neuropilin-1-linked angiogenic response patterns, spatial immunotypes associated with ICI outcome, and onco-fetal neighborhood features linked to relapse and immunotherapy response [47,61,62]. None of these classifiers, however, is yet validated as a treatment-selection biomarker. Intratumoral TLS signatures add another layer, particularly because perioperative dual-ICI data suggest an association with response and implicate a B-cell-dependent component of anti-tumor immunity [39]. These biomarkers are attractive not merely because they correlate with outcome but because they are mechanistically interpretable and may therefore inform therapeutic selection.

Blood-based biomarkers may extend this logic into longitudinal decision-making. Circulating tumor DNA (ctDNA) for minimal residual disease (MRD) detection after curative resection has emerged as one of the most rigorously evaluated blood-based signals: a recent meta-analysis of postoperative ctDNA across HCC studies found that ctDNA positivity was associated with substantially shorter recurrence-free survival (pooled HR 4.48, 95% CI 2.56–7.82) and worse overall survival (pooled HR 2.99, 95% CI 1.94–4.61) [100], and tumor-informed personalized ctDNA assays have demonstrated meaningful lead time over alpha-fetoprotein for recurrence detection in real-world cohorts [101]. A broader systematic review of circulating blood biomarkers in HCC supports this picture, with ctDNA and circulating tumor cells emerging as the most robust MRD-detection approaches, although clinical utility trials remain pending [102]. Beyond MRD, composite clinical inflammatory scores such as CRAFITY, peripheral T-cell exhaustion profiles from perioperative dual-ICI series, anti-drug antibody measurements, and gut microbiome signatures may capture complementary host- and treatment-level modifiers of response [39,92,103]. These approaches are appealing because they may permit non-invasive monitoring of dynamic biological change rather than single-timepoint classification alone.

Integrative platforms will likely be required to move beyond isolated biomarker candidates. Hypoxia scores, machine-learning-based clinical-imaging classifications such as the A–B–C framework, and multi-omic approaches incorporating single-cell and spatial transcriptomic data all point toward a more composite model of precision immunotherapy [60,98]. The task ahead is therefore not simply the discovery of additional candidate biomarkers but their prospective incorporation into biomarker-stratified combination trials. Importantly, this biomarker agenda should remain biologically grounded: the goal is not merely to improve prediction but to identify which barrier dominates in a given tumor and which therapeutic intervention is most likely to reverse it.

## 7. Conclusions

Immunotherapy has redefined the treatment of advanced HCC, yet only a subset of patients derive durable benefit, and the mechanisms of response and resistance remain incompletely understood. The available evidence supports several biologically plausible resistance pathways, but much of this evidence remains correlative or preclinical, and the roles of individual myeloid and stromal subpopulations are not yet fully established. The next phase of progress will likely depend less on adding more drugs to existing regimens than on learning to read the immune architecture of each tumor, including its dominant barriers, etiologic context, and residual capacity for immune remodeling. From a treatment design perspective, this implies a precision approach in which drug choice, disease setting, and timing of intervention are considered together across resection, locoregional therapy, and advanced disease, rather than treated as separate decisions. Disease etiology, in this context, should be regarded as an important biological and stratification variable rather than a validated standalone criterion for selecting or withholding ICI-based therapy. Realizing precision immunotherapy in HCC will require prospective, biomarker-stratified trials that test whether matching therapy to a measurable dominant barrier improves patient outcomes.

## Figures and Tables

**Table 1:** Selected major immune checkpoint inhibitor-based trials in hepatocellular carcinoma.

Trial	Setting and Regimen	Key Efficacy Result	Mechanistic Interpretation
CheckMate 459 [70]	Ph3; 1L unresectable HCC; nivolumab vs. sorafenib	OS 16.4 vs. 14.7 mo; HR 0.85 (95% CI 0.72–1.02); primary endpoint not met	PD-1 monotherapy benefits selected primed tumors but leaves upstream barriers intact.
KEYNOTE-224 [71]	Ph2; 2L post-sorafenib HCC; pembrolizumab single-arm	ORR 17%	Established baseline activity of single-agent PD-1 blockade in HCC.
KEYNOTE-240 [72]	Ph3; 2L post-sorafenib HCC; pembrolizumab vs. placebo	OS 13.9 vs. 10.6 mo; HR 0.78 (95% CI 0.61–1.00); prespecified threshold not met	Showed a clinically meaningful PD-1 signal despite statistical design limitations.
KEYNOTE-394 [73]	Ph3; 2L Asian HCC; pembrolizumab vs. placebo + BSC	OS 14.6 vs. 13.0 mo; HR 0.79 (95% CI 0.63–0.99); *p* = 0.018	Confirmed reproducible second-line PD-1 activity in HCC.
IMbrave150 [7,74]	Ph3; 1L unresectable HCC; atezolizumab + bevacizumab vs. sorafenib	Updated OS 19.2 vs. 13.4 mo; HR 0.66 (95% CI 0.52–0.85)	Anti-VEGF therapy enhances checkpoint blockade through vascular and myeloid remodeling.
HIMALAYA [8]	Ph3; 1L unresectable HCC; STRIDE vs. sorafenib	OS 16.4 vs. 13.8 mo; HR 0.78 (96.02% CI 0.65–0.93) ^a^	STRIDE supports CTLA-4 priming followed by PD-L1–pathway effector rescue.
CheckMate 9DW [9]	Ph3; 1L unresectable HCC; nivolumab + ipilimumab vs. lenvatinib/sorafenib	OS 23.7 vs. 20.6 mo; HR 0.79 (95% CI 0.65–0.96); ORR 36% vs. 13%	Dual PD-1/CTLA-4 blockade supports coordinated priming and effector rescue.
CARES-310 [75]	Ph3; 1L unresectable HCC; camrelizumab + rivoceranib vs. sorafenib	Final OS 23.8 vs. 15.2 mo; HR 0.64 (95% CI 0.52–0.79)	Extends ICI plus anti-angiogenic benefit to TKI-based vascular remodeling.
ORIENT-32 [76]	Ph2–3; 1L Asian HCC; sintilimab + IBI305 vs. sorafenib	Median OS NR vs. 10.4 mo; HR 0.57 (95% CI 0.43–0.75)	Reproduces the anti-VEGF plus PD-1 principle in predominantly HBV-related HCC.
Kaseb et al. [77]	Randomized Ph2; resectable HCC; perioperative nivolumab ± ipilimumab	Major pathologic response 33% (nivolumab)/27% (nivolumab + ipilimumab); no surgery delayed	Supports the intact tumor as an in situ antigen source before resection.
IMbrave050 [78,79]	Ph3; adjuvant high-risk HCC; atezolizumab + bevacizumab vs. surveillance	Interim RFS HR 0.72 (adjusted 95% CI 0.53–0.98); updated RFS HR 0.90 (95% CI 0.72–1.12); benefit not sustained	Highlights that advanced-disease efficacy may not translate to postoperative MRD.
COSMIC-312 [80]	Ph3; 1L unresectable HCC; atezolizumab + cabozantinib vs. sorafenib	OS 15.4 vs. 15.5 mo; HR 0.90 (96% CI 0.69–1.18) ^a^; primary endpoint not met	Suggests ICI plus TKI is not automatically synergistic; interpretation is also influenced by trial design, comparator activity, toxicity, and patient selection.
LEAP-002 [81]	Ph3; 1L unresectable HCC; lenvatinib + pembrolizumab vs. lenvatinib + placebo	OS 21.2 vs. 19.0 mo; HR 0.84 (95% CI 0.71–1.00); prespecified threshold not crossed	Illustrates the difficulty of demonstrating added ICI benefit against an active TKI backbone; interpretation should be balanced against comparator efficacy and trial design factors.

Abbreviations: 1L, first-line; 2L, second-line; BSC, best supportive care; CTLA-4, cytotoxic T-lymphocyte-associated protein 4; HBV, hepatitis B virus; HCC, hepatocellular carcinoma; HR, hazard ratio; ICI, immune checkpoint inhibitor; mo, months; MRD, minimal residual disease; ORR, objective response rate; OS, overall survival; PD-1, programmed cell death protein 1; PD-L1, programmed death-ligand 1; Ph, phase; RFS, recurrence-free survival; STRIDE, single tremelimumab regular interval durvalumab; TKI, tyrosine kinase inhibitor; VEGF, vascular endothelial growth factor. ^a^ For HIMALAYA and COSMIC-312, confidence intervals are reported at the 96.02% and 96% levels, respectively, as presented in the primary publications, reflecting the alpha allocated to these comparisons; all remaining confidence intervals are 95% intervals, including adjusted 95% intervals where reported. Note: Trials were selected to illustrate major clinical and mechanistic principles in HCC immunotherapy rather than provide an exhaustive catalog of all systemic therapy studies.

**Table 2:** Barrier-based framework for HCC immunotherapy. This table is intended as a conceptual organizing aid; dominant barriers are rarely measured prospectively, and the listed biomarkers are not validated for treatment selection unless explicitly stated. Representative evidence is discussed and cited in the corresponding sections of the text.

Dominant Barrier/Context	Representative Biology or Biomarkers	Therapeutic Rationale	Example Strategies	Evidence and Current Applicability
Checkpoint-restrained inflamed tumor	PD-1/PD-L1, IFN signaling, TCF1+ progenitor-exhausted CD8+ T cells; immune-inflamed phenotype	Reinvigorate pre-existing anti-tumor immunity	Established first-line ICI-combination practice; PD-1/PD-L1 biomarkers not routinely used for selection	Established first-line ICI-combination practice; not routinely biomarker-selected
Vascular/trafficking barrier	VEGF, abnormal vasculature, CXCL12+ TECs; low neuropilin-1 as candidate marker	Vascular normalization; improved effector-cell trafficking	Atezolizumab/bevacizumab; ICI + TKI; investigational CXCL12/PD-1 co-targeting	Established regimen class; specific biomarkers not validated for selection
Myeloid–stromal exclusion	SPP1+ TAMs, TREM2+/CD14+ myeloid cells, POSTN+ CAFs, TGF-β; CD48+ TAM program	Relieve exclusion; reprogram suppressive myeloid–stromal niche	Anti-VEGF/TKI; TGF-β-, CSF1R-, CCR2/5-directed agents; CD48-directed strategies	Mostly translational/investigational; not validated for selection
Priming/antigen-presentation failure	Low cDC1, impaired antigen presentation, low tumor mutational burden	Increase antigen release and cross-priming	TACE, SBRT, ablation, or HAIC + ICI; vaccines; oncolytic viruses	Mixed clinical/translational; setting-specific or investigational
Hypoxia–metabolic suppression	HIF-1α, adenosine axis, lactate, IDO1	Improve perfusion; relieve metabolic suppression	Anti-VEGF; adenosine-axis inhibitors; metabolic-targeting agents	Mostly translational; investigational
Tumor-cell-autonomous immune exclusion	CTNNB1/Wnt or AXIN1 activation; impaired chemokine/cDC1 recruitment	Restore immune entry or priming	Wnt/β-catenin-directed approaches	Candidate predictive biomarker; not routine for treatment selection; therapies investigational
Etiology-conditioned immune ecology	Viral, MASLD/MASH, and alcohol-related immune contexture	Stratify biology and resistance mechanisms	Etiology-aware trial design and biomarker development	Heterogeneous evidence; not a standalone ICI selection biomarker
Adaptive treatment-induced resistance	LAG-3, TIM-3, TIGIT; therapy-induced stromal rewiring	Target emergent inhibitory pathways when dominant	Next-generation checkpoint combinations	Mixed/early clinical; investigational; dominance must be demonstrated

Abbreviations: AXIN1, axis inhibition protein 1; CAF, cancer-associated fibroblast; CCR2/5, C-C chemokine receptor 2/5; CD8, cluster of differentiation 8; CD14, cluster of differentiation 14; CD48, cluster of differentiation 48; cDC1, conventional type 1 dendritic cell; CSF1R, colony-stimulating factor 1 receptor; CXCL12, C-X-C motif chemokine ligand 12; HAIC, hepatic arterial infusion chemotherapy; HCC, hepatocellular carcinoma; HIF-1α, hypoxia-inducible factor-1α; ICI, immune checkpoint inhibitor; IDO1, indoleamine 2,3-dioxygenase 1; IFN, interferon; LAG-3, lymphocyte-activation gene 3; MASLD/MASH, metabolic dysfunction-associated steatotic liver disease/steatohepatitis; PD-1, programmed cell death protein 1; PD-L1, programmed death-ligand 1; POSTN, periostin; SBRT, stereotactic body radiation therapy; SPP1, secreted phosphoprotein 1; TACE, transarterial chemoembolization; TAM, tumor-associated macrophage; TCF1, T-cell factor 1; TEC, tumor-associated endothelial cell; TGF-β, transforming growth factor beta; TIGIT, T-cell immunoreceptor with Ig and ITIM domains; TIM-3, T-cell immunoglobulin and mucin domain-containing protein 3; TKI, tyrosine kinase inhibitor; TREM2, triggering receptor expressed on myeloid cells 2; VEGF, vascular endothelial growth factor.

## Data Availability

No new data were created or analyzed in this study. Data sharing is not applicable to this article.
